# A Priority-Aware Multi-Agent Reinforcement Learning Framework for Collaborative Intelligent Sensing in Social IoT

**DOI:** 10.3390/s26165298

**Published:** 2026-08-21

**Authors:** Jing Zhu

**Affiliations:** School of Computer and Information Science, Qinghai Institute of Technology, Xining 810016, China; jzhu@qhit.edu.cn

**Keywords:** collaborative intelligent sensing, Social Internet of Things, priority-aware resource coordination, multi-agent Q-learning, quality of experience (QoE)

## Abstract

Collaborative intelligent sensing in the Social Internet of Things (Social IoT) relies on distributed AI-enabled sensors to support complementary information sharing, multimodal perception, and real-time autonomous decision-making. Under high-load conditions, mismatches between resource provisioning and sensing quality of experience (QoE) can significantly degrade system performance in applications such as smart cities. To address this issue, this paper proposes a service priority-aware collaborative sensing support framework based on a joint next-generation passive optical network (NG-PON) and cooperative intelligent service-based radio access network (CIS-RAN) architecture. The framework enables edge AI-driven inference and distributed sensor collaboration in heterogeneous Social IoT environments. Service-slice-specific priority weights are assigned to optical network units (ONUs) and wavelengths according to the QoE requirements and latency sensitivity of sensing tasks, allowing dynamic wavelength tuning that prioritizes high-impact collaborative services. The utility of a centralized intelligent processing pool is formulated to achieve priority-consistent and efficient resource coordination under collaborative constraints. In addition, a multi-agent AI-driven optimization framework is employed to derive adaptive resource allocation strategies that incorporate service priorities while satisfying stringent service-level agreements (SLAs). Simulation results show that the proposed framework improves system-level proxy metrics, including total utility, wavelength satisfaction, and resource utilization, compared with representative baseline schemes.

## 1. Introduction

To meet the growing demands for massive connectivity, pervasive sensing, and heterogeneous services in Social Internet of Things (Social IoT) environments, traditional radio access network (RAN) architectures must evolve. Such transformation enables collaborative intelligent sensing among distributed artificial intelligence (AI)-powered sensors, which form goal-oriented networks for complementary information sharing, multimodal perception, and real-time autonomous decision-making in applications such as smart cities and digital twins [1,2].

Beyond cloud-centric centralized RAN (C-RAN), research is moving towards cooperative, intelligent, and service-based RAN (CIS-RAN). CIS-RAN features native AI, pervasive cooperation among network entities, and a flexible service-based design. It aims to achieve holistic network autonomy, optimal resource allocation, and the extension of cooperation from communication to sensing and AI-driven decision-making.

The adoption of CIS-RAN imposes unprecedented demands on the fronthaul network, including ultra-low deterministic latency, high capacity, and intelligent programmability, to efficiently interconnect distributed sensor networks across heterogeneous Social IoT environments. Next-generation passive optical networks (NG-PONs), evolving beyond time- and wavelength-division multiplexed PON (TWDM-PON) with technologies such as 50G-PON, WDM-PON, and their coherent variants, are pivotal in fulfilling these demands, providing the necessary bandwidth and flexibility to support CIS-RAN’s dense cooperation and functional splits for collaborative intelligent sensing [3,4]. The convergence of an intelligent, slice-aware optical fronthaul with AI-native, service-based CIS-RAN creates a powerful symbiotic architecture that serves as key underlying infrastructure enabling edge AI-driven perception and inference in heterogeneous Social IoT scenarios.

Managing tightly coupled computational, spectral, and connectivity resources in such an integrated environment remains a critical challenge for enabling complementary information sharing and real-time autonomous decision-making in collaborative intelligent sensing systems [5,6]. Resource demands from service-based network functions within the CIS-RAN processing pool are highly dynamic and spatially uneven, and static provisioning leads to severe inefficiency [7,8]. Consequently, designing autonomous, context-aware, and cooperative resource management mechanisms is paramount to enable efficient collaborative intelligent sensing and edge AI-driven inference across heterogeneous sensing tasks.

Prior research has laid important groundwork in resource abstraction [9], economic models [10], and fairness [11]. Recognizing service heterogeneity, quality of service/quality of experience (QoS/QoE)-aware schemes have been proposed in [12,13,14,15]. For example, the authors of [13] addressed resource allocation in multi-tenant and multi-service 5G network slicing, modeling the maximization of system spectral efficiency as an NP-hard combinatorial mixed-integer nonlinear programming problem and proposing a polynomial time swarm natural search algorithm combined with power and subcarrier allocation. In [14], a novel machine learning–enhanced classification scheme was designed for interference management and offloading, categorizing users by interference types and levels via multi-binary classification to improve service QoE and resource utilization in 5G heterogeneous networks. In [15], solutions were proposed for accurate user localization, multi-point/multi-edge pairing optimization, and dynamic power allocation in non-orthogonal multiple access (NOMA) systems, along with a mathematical model adapted to user demand differences, effectively improving system performance. However, these works often lack the intrinsic cooperative intelligence and end-to-end service-based orchestration envisioned for CIS-RAN, particularly in supporting distributed sensor collaboration for Social IoT.

The above concepts are interconnected within a unified architectural vision. Social IoT provides the application context where distributed AI-powered sensors establish social relationships to enable collaborative sensing. CIS-RAN serves as the service-based radio access infrastructure that natively supports AI-driven cooperation and edge intelligence. NG-PON provides the high-capacity, reconfigurable optical transport layer that interconnects distributed sensors with the edge processing pool. Edge AI enables real-time inference and autonomous decision-making at the network edge. The proposed framework bridges these elements by formulating service priority-aware resource allocation as a cross-domain optimization problem that jointly considers optical wavelength assignment and computational resource partitioning, guided by QoE-driven priority metrics derived from heterogeneous CIS requirements.

To address this convergence gap, we propose a novel service priority-aware collaborative sensing support mechanism for the joint NG-PON and CIS-RAN architecture. This mechanism serves as a foundational technology to enable efficient collaborative intelligent sensing in Social IoT environments. The main contributions of this work are summarized as follows:

(1) A QoE-driven service-slice weight model is established to map end-to-end requirements into unified priority weights at both optical network unit (ONU) and wavelength levels. ONU-level weights enforce intra-wavelength service differentiation, while aggregated wavelength-level weights guide inter-wavelength coordination. This approach translates heterogeneous QoE constraints from wireless and optical domains into consistent prioritization rules for resource allocation in distributed sensor networks supporting multimodal perception and complementary information sharing.

(2) The cross-domain resource allocation task is formulated as a cooperative utility maximization problem over the shared intelligent processing pool, where pooled compute resources are partitioned among wavelengths under global feasibility constraints. Weighted concave utilities reflect service priorities and fairness, and joint decisions on wavelength tuning and compute partitioning are captured in a single optimization framework, embodying CIS-RAN’s collective optimization principle to facilitate edge AI-driven inference and cooperative intelligence in heterogeneous Social IoT sensing scenarios.

(3) A multi-agent AI-enhanced resource allocation algorithm is developed, modeling each wavelength entity as an agent in a cooperative learning paradigm to determine tuning strategies. Through weight-aware rewards and coordinated updates, the global utility is optimized under limited pool capacity and time-varying service composition, achieving higher QoE satisfaction for high-priority services while maintaining overall resource efficiency compared to non-cooperative baselines.

In summary, the key innovation of this work lies in the unified integration of QoE-driven dynamic priority modeling, cross-domain cooperative bargaining formulation, and multi-agent reinforcement learning within a joint NG-PON and CIS-RAN architecture, specifically designed to support CIS in Social IoT. Unlike prior works that address optical resource allocation or RAN compute management in isolation, the proposed service priority-aware resource allocation (SPRA) framework achieves end-to-end priority-consistent resource coordination across heterogeneous domains.

The remainder of this paper is organized as follows. The system model is described in Section 2. The resource allocation algorithm is introduced in Section 3. Simulations are presented in Section 4, and Section 5 concludes the entire paper.

### Related Works

A substantial body of literature has laid the groundwork for resource management in the constituent technologies of our proposed architecture, yet a significant gap remains in their holistic integration for collaborative intelligent sensing in Social IoT.

In next-generation PONs, research has advanced beyond static TWDM-PON, with recent efforts focusing on slice-aware bandwidth allocation and dynamic wavelength assignment to support heterogeneous traffic. SDN controllers enable agile provisioning [16], while advanced QoS-aware schemes employ queueing theory or heuristics to prioritize classes such as EF and AF [17]. However, most approaches treat the PON as an isolated transport layer, basing allocation on local buffer states or basic SLAs, without real-time awareness of RAN processing pool demands and cooperative dynamics. This decoupling results in suboptimal end-to-end performance.

The evolution from C-RAN to CIS-RAN introduces new architectural principles. While C-RAN centralizes processing for cooperative radio techniques and statistical multiplexing gains, it suffers from stringent fronthaul latency/bandwidth constraints and computationally heavy centralized resource management [18,19]. The emerging CIS-RAN paradigm, central to 6G research, incorporates native AI, pervasive cooperation, and service-based design [20,21]. Initial efforts explore AI-driven resource prediction and allocation in the RAN intelligent controller [22]. However, resource allocation in full-fledged CIS-RAN remains nascent, with most proposals confined to the wireless/compute domain and treating the fronthaul as having a fixed capacity. Cross-domain cooperative optimization—leveraging NG-PON wavelength reconfigurability to balance processing pool utility—has received insufficient attention.

The application of artificial intelligence, particularly reinforcement learning (RL), to network resource allocation has grown significantly. Model-free algorithms like Q-learning are widely used for optimal policy learning in dynamic environments [23]. Multi-agent reinforcement learning (MARL) extends this to distributed settings, enabling cooperative or competitive behaviors among entities such as base stations or slices [24,25]. Recent MARL variants have been applied to edge task offloading in Internet of Vehicles sensing with prioritized experience replay [26]. However, these approaches are typically confined to siloed domains and their use in tightly coupled, multi-resource environments like the joint NG-PON-CIS-RAN architecture remains largely unexplored. The proposed SPRA algorithm adopts tabular Q-learning for its convergence under standard assumptions, interpretability, and lightweight deployment, while we discuss scalability pathways through function approximation for larger configurations.

Finally, game-theoretic frameworks naturally model strategic interactions in resource sharing. Bargaining games, particularly the generalized Nash bargaining solution, are well suited for cooperative scenarios, yielding fair and Pareto-optimal outcomes [27]. Prior applications include resource allocation in cloud computing and virtual networks [28,29], while security and privacy proposals address fine-grained intrusion protection in distributed settings [30]. However, these models have not been integrated with online learning for dynamic wavelength-and-compute co-allocation in Social IoT contexts. Moreover, limited attention has been given to system-level coordination mechanisms that preserve collaborative sensing QoE across tightly coupled optical and RAN domains in Social IoT scenarios. Unlike existing Nash bargaining approaches for cloud resource management, our framework uniquely combines GNBS-based allocation with MARL-based wavelength tuning, achieving both theoretical fairness guarantees and adaptive online optimization.

Although the above studies provide useful foundations for resource allocation in optical fronthaul, RAN slicing, and learning-based optimization, most of them focus on a single domain, such as wireless resource allocation, edge computing, or optical transport. Existing MARL-based schemes mainly optimize spectrum, power, or task offloading decisions, while the coupling between NG-PON wavelength tuning and CIS-RAN processing pool allocation is rarely considered. In addition, many deep learning–based approaches improve prediction or decision accuracy but usually require large-scale training data and have limited interpretability for priority-aware service coordination. Therefore, a lightweight and interpretable resource coordination framework that jointly considers service priorities, wavelength reconfiguration, and processing pool utility remains insufficiently explored.

## 2. System Model

### 2.1. Integrated NG-PON and CIS-RAN Architecture for Collaborative Intelligent Sensing in Social IoT

**Figure 1:**
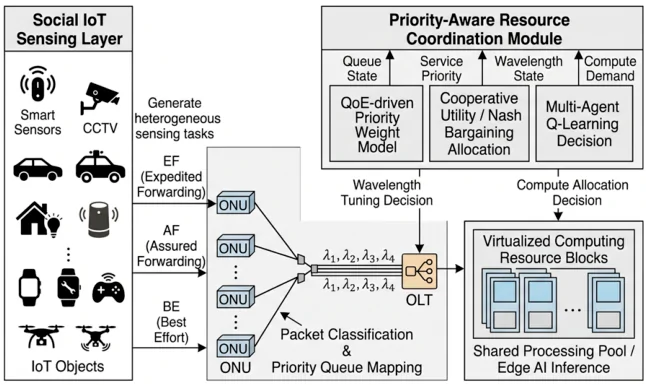
Conceptual workflow of the proposed priority-aware SPRA framework.

To provide an intuitive overview of the proposed framework, Figure 1 illustrates the conceptual workflow of the priority-aware SPRA mechanism. Heterogeneous Social IoT sensing tasks are first classified into EF, AF, and BE services on the ONU side. Then, the priority-aware resource coordination module jointly considers queue states, service priorities, wavelength states, and compute demands to determine wavelength tuning and compute allocation decisions [31]. The resulting decisions are applied to the NG-PON fronthaul and the shared CIS-RAN processing pool to support edge AI inference and priority-consistent collaborative sensing.

**Figure 2:**
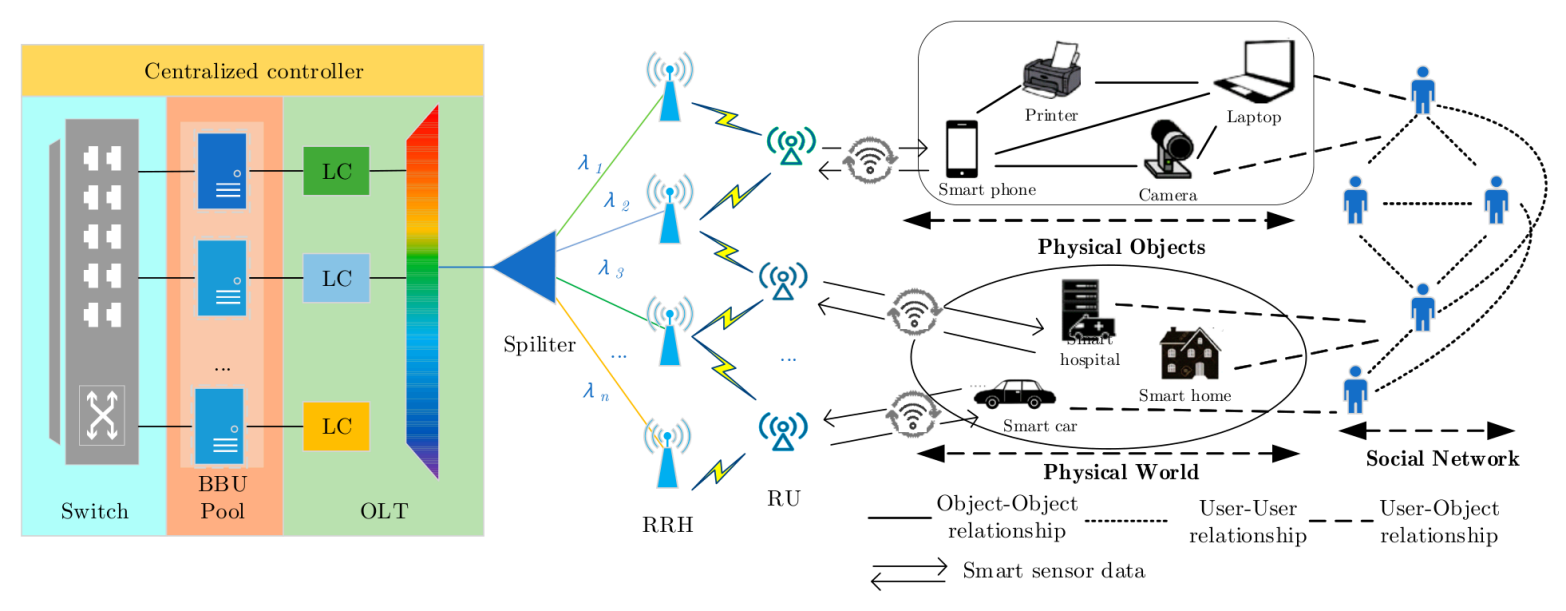
Joint NG-PON and CIS-RAN architecture for collaborative intelligent sensing in Social IoT environments.

This section describes the proposed joint NG-PON and CIS-RAN system model, which integrates optical transport and service-based RAN functions to support collaborative intelligent sensing in Social IoT environments. As illustrated in Figure 2, a programmable optical line terminal (OLT) is co-located with an edge-cloud RAN processing pool, which hosts disaggregated CU/DU functions as cloud-native microservices coordinated by a native AI orchestrator. The OLT supports K tunable upstream wavelengths via software-defined transceivers, enabling dynamic wavelength reconfiguration. ONUs connected to the OLT through a shared PON tree aggregate data from multiple radio units (RUs), which collect multimodal sensing data from distributed AI-powered sensors. Aggregated sensing data is forwarded through the PON to the processing pool for real-time edge analysis and decision-making, enabling information sharing and collaborative intelligent sensing. The system operates in basic transmission intervals, with wavelength and computational resource adjustments performed at each control interval to meet dynamic service demands in heterogeneous Social IoT scenarios. The main notations are summarized in Table 1.

**Figure 3:**
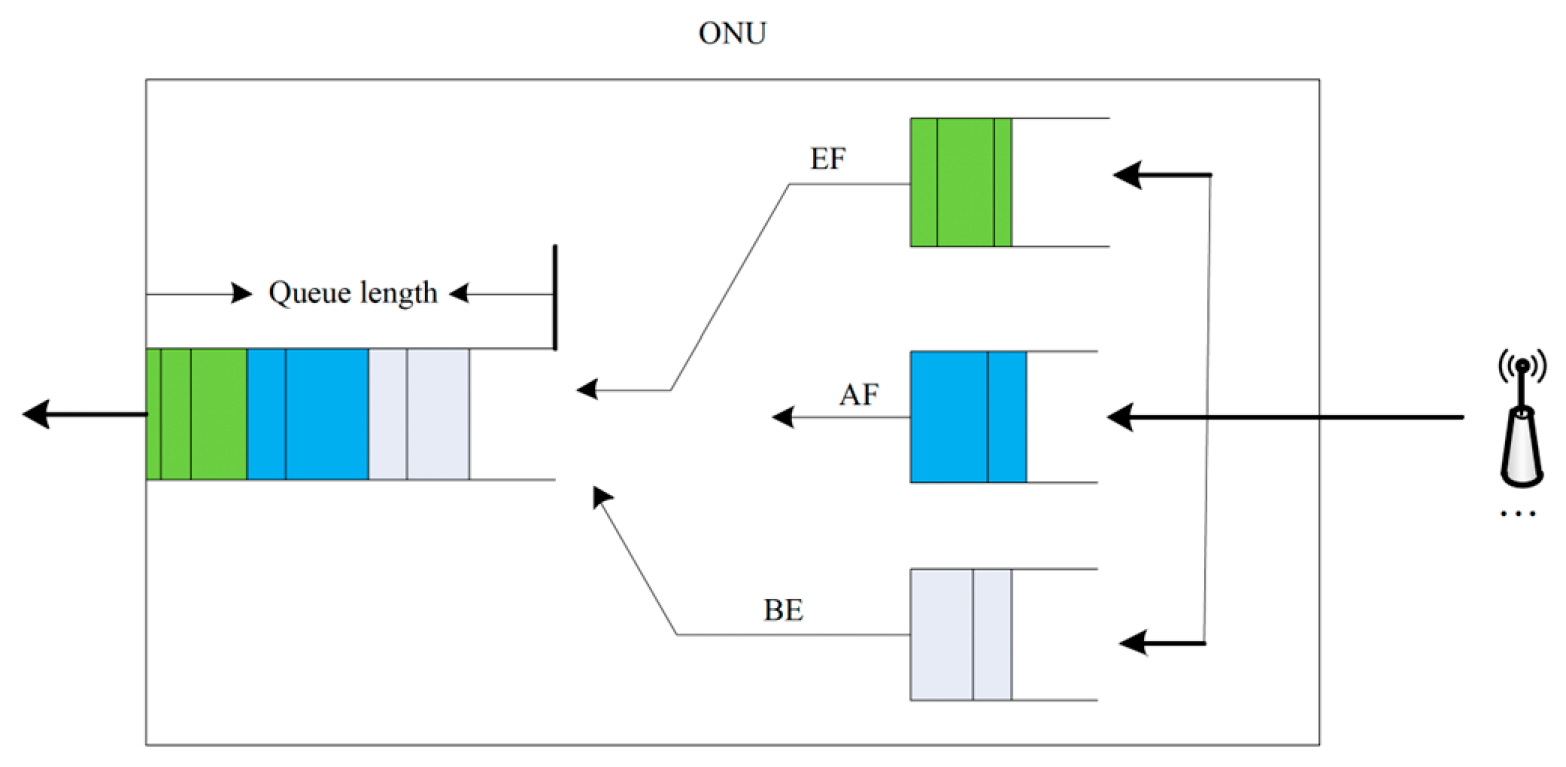
Two-level buffer queue.

Since service taxonomies in the wireless domain and the optical transport domain differ significantly, each ONU implements a two-level buffering structure, as shown in Figure 3, to bridge heterogeneous QoS semantics and ensure end-to-end QoE consistency. Arriving packets from RUs are first classified in the wireless domain into service classes based on their QoS descriptors (e.g., delay budget, reliability requirement, and packet size). Each packet is marked with a priority label p(s) and inserted into the corresponding wireless-side sub-queue. A traffic-shaping module at the ONU then maps the wireless-side arrival process into optical-side burst/packet scheduling by (i) smoothing short-term rate fluctuations, (ii) enforcing per-class rate constraints using token-bucket or leaky-bucket mechanisms, and (iii) generating optical transmission requests compatible with upstream wavelength scheduling.

On the optical side, packets are re-mapped into a unified three-class forwarding model aligned with transport-layer QoS treatment: expedited forwarding (EF), assured forwarding (AF), and best effort (BE). EF aggregates latency-critical sensing traffic (real-time perception tasks) and is served with strict priority and bounded queueing latency; AF aggregates throughput-guaranteed sensing traffic (collaborative inference tasks) and is served with minimum-rate assurance plus controlled loss; BE collects elastic sensing traffic (background monitoring tasks) and utilizes the remaining capacity opportunistically. The mapping from wireless classes to {EF,AF,BE} is realized by a deterministic policy ϕ:S→{EF,AF,BE}, complemented by per-class shaping parameters that regulate admission control and burstiness. During upstream scheduling, the ONU transmits optical-domain packets in non-increasing priority order (EF≻AF≻BE), while the shaping stage ensures high-priority traffic protection from starvation and maintains consistent end-to-end QoS targets across the wireless–optical boundary [32].

In the physical network, remote radio heads (RRHs) forward received sensor data to their directly connected ONUs. The ONUs utilize the common public radio interface (CPRI) or its next-generation equivalents (e.g., eCPRI) to transmit user and sensing information to the OLT and the centralized processing pool. The native AI orchestrator in the pool analyzes this aggregated data, performs edge AI-driven inference for collaborative sensing tasks, allocates wavelengths and computational resources to the ONUs based on service priorities, and disseminates configuration commands to the underlying physical network for implementation. This cross-domain orchestration enables efficient resource utilization while supporting knowledge inference and cooperative intelligence among distributed sensors in Social IoT networks.

### 2.2. QoE-Driven Priority Weight Model

In Social IoT environments, different network slices exhibit vastly diverse QoE requirements due to heterogeneous collaborative sensing tasks, such as real-time multimodal perception, complementary information sharing, and autonomous decision-making among distributed AI-powered sensors. To enable service priority-aware resource allocation across the joint NG-PON and CIS-RAN architecture, we establish a dynamic weight model that maps real-time slice intents and contextual network states into unified priority metrics at both ONU and wavelength levels.

Denote the set of data packets, ONUs, and active wavelengths as P, O, and L, respectively, where $\left|P\right|=P$, $\left|O\right|=O$, and $\left|L\right|=L$. Let $q_{i}$ denote the service-priority weight of packet i, which is determined by its slice type, QoE target, and current service requirement. The weight of ONU j at time t is given as follows:

(1) $$w_{j}^{\mathrm{ONU}}(t)=\frac{\sum_{i\in P}\alpha_{i,j}(t)q_{i}}{\sum_{i\in P}\alpha_{i,j}(t)},$$

where $\alpha_{i,j}(t)=1$ represents the time when ONU *j* provides service to data packet *i*; otherwise,$\alpha_{i,j}(t)=0$. Let $\beta_{j,k}(t)\in \{0,1\}$ represent the wavelength currently used by ONU *j*. When the wavelength of ONU *j* is *k*, $\beta_{k,j}(t)=1$; otherwise, $\beta_{k,j}(t)=0$. The weight of wavelength *k* in the network at time *t* is obtained as follows:

(2) $$w_{k}^{\mathrm{WL}}(t)=\frac{\sum_{j\in O}\beta_{j,k}(t)w_{j}^{\mathrm{ONU}}(t)}{\sum_{m\in L}\sum_{j\in O}\beta_{j,m}(t)w_{j}^{\mathrm{ONU}}(t)},$$

where the $w_{k}^{\mathrm{WL}}(t)$ value of ranges between (0,1), reflecting the average QoS demand of the services carried by wavelength k at time t. Let $R_{k}(t)$ denote the computational resources allocated to wavelength k at time t. Then the computational resources allocated to wavelength *k* at time t can be expressed as follows:

(3) $$R_{k}(t)=w_{k}^{\mathrm{WL}}(t)\times C\left(t\right).$$

The intelligent processing pool is modeled as a shared resource substrate hosting service-based RAN functions and their AI assistants. In practice, these functions may be instantiated as cloud-native microservices (containers) connected through a service mesh. Compute capacity is heterogeneous (CPU cores, memory bandwidth, accelerators), and strict isolation is typically enforced through virtualization primitives (cgroups/quotas) and admission control. To keep the optimization tractable while preserving cross-domain coupling, we abstract the processing pool into a finite number of virtualized resource blocks (RBs), which directly impacts the inference completion time of sensing tasks. One RB represents a normalized compute unit that can be consumed by a wavelength according to its aggregate packet workload. Let *C*(*t*) denote the total RB budget at time *t*. The controller allocates $C_{k}\left(t\right)$ to wavelength *k* such that $\sum_{k}C_{k}\left(t\right)=C\left(t\right)$. Within each wavelength k, the assigned RBs are further distributed to its associated ONUs using local scheduling mechanisms (e.g., weighted fair queuing guided by slice-specific priorities), thereby ensuring efficient support for edge AI-driven inference and cooperative intelligence across distributed sensor networks in Social IoT.

### 2.3. Service Priority-Aware Collaborative Sensing Support Mechanism

In the joint NG-PON and CIS-RAN architecture, the central controller makes two coupled decisions per control interval: (i) wavelength tuning of ONUs and (ii) compute allocation from the shared processing pool. Each ONU i∈O maintains class-tagged buffers; from the buffered packets, the controller derives a per-ONU compute request $d_{i}\left(t\right)$ and a service-priority weight $q_{i}$ (larger $q_{i}$ indicates tighter QoS). For wavelength k∈L, define the ONU group $O_{k}\left(t\right)$ after tuning, while the aggregate compute demand is $D_{k}\left(t\right)=\sum_{j\in O_{k}\left(t\right)}\alpha_{i,j}\left(t\right)d_{i}\left(t\right)$. Let C be the total compute capacity in the pool and $c_{k}$ the compute share assigned to wavelength *k*, with $\sum_{k\in L}c_{k}\leq C$.

If $\sum_{i\in O}d_{i}\left(t\right)\leq C\left(t\right)$, the allocation is demand-matching, i.e., $c_{k}=D_{k}$, and each ONU receives its request. Otherwise, the controller solves a weighted bargaining allocation across wavelengths. Specifically, each wavelength *k* is modeled as a player whose utility is an increasing, concave function of its allocated compute share, e.g., as follows:

(4) $$U_{k}(c_{k})=w_{k}^{\mathrm{WL}}(t)\cdot \log(1+\alpha c_{k}),$$

where α>0 controls marginal utility. The compute partition $c_{k}$ is then obtained by maximizing the weighted Nash product under capacity constraints:

(5) $$\begin{array}{l} \max_{\left\{c_{k}\geq 0\right\}}\prod_{k\in L}(U_{k}(c_{k})-U_{k}^{\mathrm{dis}}{)}^{\beta_{k}}\\ s.t.\;\sum_{k}c_{k}\leq C, \end{array}$$

where $U_{k}^{\mathrm{dis}}$ is the disagreement utility and $\beta_{k}$ is the bargaining power. After computing $c_{k}$, the controller distributes $c_{k}$ among ONUs in $O_{k}$ using the same priority rule, e.g., proportional to $q_{i}d_{i}\left(t\right)$. This converts the pool utility maximization with heterogeneous QoS into a cooperative bargaining problem whose solution is feasible and priority-consistent.

The choice of logarithmic utility $U_{k}\left(t\right)$ is motivated by three considerations. First, the logarithmic form is strictly concave and monotonically increasing in $R_{w}$, satisfying the regularity conditions required for a well-defined Nash bargaining solution. Second, logarithmic utility captures diminishing marginal returns: the first few resource blocks allocated to a wavelength yield substantial performance improvement, while additional blocks provide progressively smaller gains, which is consistent with practical resource allocation behavior where over-provisioning offers little benefit. Third, the resulting allocation achieves proportional fairness, a well-established fairness criterion in network resource management [11,27]. Alternative utility forms include linear utility (which yields max-sum allocation without fairness consideration), sigmoidal utility (which captures threshold effects but introduces non-convexity), and the α-fair family $U\left(R\right)=R^{1-\alpha }/(1-\alpha )$ for α > 0, which generalizes our formulation (α = 1 recovers logarithmic utility). We select the logarithmic form as it offers the best balance between theoretical tractability and practical relevance.

### 2.4. Objective Function

Let $c_{k}^{\min}$ denote the minimum computational capability required by data packet i per transmission time interval. Then the minimum computational resource required by wavelength k is calculated as follows:

(6) $$c_{k}^{\min}\left(t\right)=\sum_{j\in O}\sum_{i\in P}\alpha_{i,j}(t)\beta_{j,k}(t)c_{i}^{\min},$$

where $c_{i}^{\min}$ is the minimum compute requirement of packet i. Based on the weight of wavelength k at time t, the minimum required computational resource, and the obtainable computational resource, the utility function for the computational resources acquired by wavelength k at time t is defined as follows:

(7) $$U_{k}(t)=w_{k}^{\mathrm{WL}}(t)\cdot \log\left(\left(R_{k}\left(t\right)-c_{k}^{\min}\left(t\right)\right)+1\right).$$

Each ONU evaluates its preference for a solution through the wavelength utility function. During bargaining, each ONU attempts to obtain more computational resources to gain higher utility. Since $U_{k}\left(t\right)$ is strictly concave and monotonically increasing in $R_{k}\left(t\right)$, each wavelength benefits from obtaining more computational resources, but with diminishing marginal returns. The central controller must determine the optimal allocation vector $R^{*}\left(t\right)\;=\;\left[R_{1}^{*}\left(t\right),\;R_{2}^{*}\left(t\right),\;\ldots ,\;R_{K}^{*}\left(t\right)\right]$ that balances efficiency and fairness across all wavelengths under a shared resource budget.

To derive the allocation mechanism, we first consider the social welfare maximization approach, which seeks to maximize the sum of utilities: $\max\sum_{k}U_{k}(t)=\max\sum_{k}w_{k}^{\mathrm{WL}}(t)\cdot \log(1+R_{k}\left(t\right)-c_{k}^{\min}\left(t\right))$, subject to $\sum_{k}R_{k}(t)\leq C(t)$ and $R_{k}(t)\geq c_{k}^{\min}(t)$. Applying the Karush–Kuhn–Tucker (KKT) conditions, the Lagrangian is $L=\sum_{k}w_{k}^{\mathrm{WL}}(t)\cdot \ln\left(R_{k}(t)-c_{k}^{\min}(t)+1\right)-\lambda \left(\sum_{k}R_{k}(t)-C(t)\right)$. Setting $\partial L/\partial R_{k}=0$ yields $w_{k}^{\mathrm{WL}}(t)/\left(R_{k}(t)-c_{k}^{\min}(t)+1\right)=\lambda$, which gives the water-filling solution: $R_{k}^{*}(t)=c_{k}^{\min}(t)+w_{k}^{\mathrm{WL}}(t)/\lambda -1$, where *λ* is determined by the capacity constraint $\sum_{k}R_{k}^{*}(t)=C(t)$. This closed-form solution demonstrates that wavelengths with higher priority weights $w_{k}^{\mathrm{WL}}$ receive proportionally more resources beyond their minimum requirements.

A feasible solution must satisfy the following constraints: (1) the actual allocated computational resources of the system must not exceed the total computational resources in the processing pool; (2) the actual allocated computational resources of the system must meet the minimum computational resource requirement of each wavelength; (3) the computational resources allocated by the central controller must not exceed the computational resource demand of the wavelength; and (4) each ONU in the network must have a wavelength serving it.

The maximization of the processing pool’s utility can be treated as finding a generalized Nash bargaining solution (GNBS). GNBS serves as the unique fair Pareto-optimal solution among all feasible solutions, satisfying Nash’s axioms, which any reasonable solution should possess [33]. The utility of the current wavelength *k* depends on its own weight and the allocated computational resources. To achieve efficient allocation of processing pool resources, the objective function is defined as the problem of maximizing the total utility of the processing pool:

(8) $$\max\;U(t)=\sum_{k\in L}U_{k}\left(t\right).$$

The problem of maximizing the total utility of the processing pool includes the following constraints:

(9) $$\alpha_{i,j}(t)\in \{0,1\},\forall i\in P,\forall j\in O,$$

(10) $$\beta_{j,k}(t)\in \{0,1\},\forall j\in O,\forall k\in L,$$

(11) $$\sum_{k\in L}R_{k}(t)\leq C\left(t\right),$$

(12) $$0<\sum_{j\in O}\sum_{i\in P}\alpha_{i,j}(t)\leq 1,$$

(13) $$0<\sum_{k\in L}\beta_{j,k}(t)\leq 1,$$

(14) $$0<\sum_{k\in L}\chi_{k}\leq L,$$

where constraint (11) indicates that the allocated computational resources cannot exceed the total resources of the processing pool, (12) states that an ONU can use one and only one wavelength at the same time, (13) constrains that a data packet i exists in only one ONU at time t, (14) ensures that at least one wavelength is enabled in the network, and the number of enabled wavelengths cannot exceed the total number of wavelengths in the network. $\chi_{k}\in \{0,1\}$ is the enabling state of wavelength k, where $\chi_{k}=1$ if wavelength k is active. From a sensing perspective, this formulation ensures priority-consistent support for latency-critical collaborative inference tasks.

**Remark** **1** **(Scope of GNBS Existence and Uniqueness).** *For a fixed ONU-to-wavelength assignment* $\left\{\beta_{j,k}(t)\right\}$*, the GNBS-based compute-allocation subproblem admits a unique solution under the following assumptions: (i) the feasible region is non-empty, i.e.,* $C(t)\geq \sum_{k\in L}c_{k}^{\min}(t)$*; (ii) each wavelength has a feasible utility gain, i.e.,* $U_{k}(t)>U_{k}^{\mathrm{dis}}(t)$*; and (iii) the utility function is continuous, monotonically increasing, and strictly concave with respect to the allocated compute resource* $R_{k}\left(t\right)$*. Under these assumptions, the feasible set of* R(t) *is bounded and convex, and the weighted Nash bargaining solution is unique for the continuous compute-allocation subproblem.*

It should be emphasized that this result applies only to the GNBS-based compute-allocation module under a fixed wavelength assignment. The complete SPRA framework further includes discrete ONU-to-wavelength tuning and online Q-learning. Therefore, no global optimality or uniqueness guarantee is claimed for the overall mixed discrete-continuous decision process.

Coupling between GNBS Allocation and RL-Based Wavelength Tuning: The proposed SPRA framework follows a two-level decision structure. The GNBS module solves the continuous compute-allocation subproblem under a given ONU-to-wavelength assignment, while the Q-learning module learns the discrete wavelength-tuning decisions over time. Specifically, at each control interval, the current ONU-to-wavelength assignment determines the wavelength-level traffic demand $D_{k}\left(t\right)$ and weight $w_{k}^{\mathrm{WL}}(t)$. Given these quantities, the GNBS module computes the compute-resource allocation $R_{k}\left(t\right)$ and the corresponding utility $U_{k}(t)$. The resulting change in total utility is then used as the reward signal for the Q-learning module. In this way, GNBS provides fairness-aware and priority-consistent resource allocation, while Q-learning adaptively updates wavelength associations according to traffic dynamics and utility feedback.

This design avoids directly solving the full mixed-integer resource allocation problem at each control interval. Instead, it decomposes the original problem into a continuous GNBS-based compute-allocation subproblem and a discrete RL-based wavelength-tuning subproblem. Therefore, the theoretical fairness and uniqueness properties apply to the GNBS subproblem, whereas the performance of the complete SPRA framework is evaluated empirically through simulations.

## 3. Multi-Agent Q-Learning for Collaborative Intelligent Sensing

Based on the two-level structure described in Section 2.4, the role of reinforcement learning is not to replace the GNBS allocation, but to learn effective wavelength-tuning decisions over time. At each control interval, the current ONU-to-wavelength assignment determines the wavelength-level traffic demand and priority weights. Given this assignment, the GNBS module computes the corresponding compute allocation and total utility. The resulting utility improvement is then used as the reward signal for Q-learning, so that wavelength agents gradually learn which ONU migration actions can improve long-term resource efficiency and priority-aware service support.

Reinforcement learning (RL) enables agents to learn optimal policies through interaction with a dynamic environment to maximize cumulative rewards [34]. In the joint NG-PON and CIS-RAN architecture, wavelength resources and computational capacity in the centralized intelligent processing pool are limited and tightly coupled. Moreover, the global utility is directly influenced by wavelength assignment decisions. Therefore, wavelength tuning can be naturally modeled as a finite-state Markov decision process (MDP) [35], where multiple wavelengths act as cooperative agents to maximize the total processing pool utility while supporting collaborative intelligent sensing in Social IoT.

With the restricted action design and compact state features, the effective state-action space remains manageable for the considered PON configurations. Tabular Q-learning is adopted because it is lightweight and interpretable. Its convergence can be established under standard assumptions, including finite state-action spaces, bounded rewards, sufficient exploration, and appropriate learning-rate decay. In this work, the Q-learning module is used as an online adaptation mechanism, and the performance of the complete SPRA framework is evaluated empirically.

Define $S=\{s_{1},\ldots ,s_{n}\}$ as the state set of wavelengths carrying ONUs, representing the current state of wavelengths carrying ONUs in the network. If the state of a wavelength carrying ONUs at time t is s, then the state-value function of this wavelength at time t can be expressed as follows:

(15) $$V(s)=Ε\left[\sum_{t=0}^{T-1}\gamma^{t}U(s)|S_{0}=s\right].$$

The state-value function V(s) is an expected value, where the discount factor γ∈[0,1), representing the influence of current utility on long-term utility. Define the action space of a wavelength at a certain moment, which includes two basic actions: (1) a wavelength transfers one ONU from its current carrying state to another wavelength; (2) the wavelength maintains its current carrying state unchanged. According to the RL framework, if a wavelength is in state s at time t and takes action a, then the action-value function of this wavelength at time t is as follows:

(16) $$V(s,a)=Ε\left[\sum_{t=0}^{T-1}\gamma^{t}U(s,a)|S_{0}=s\right].$$

State and action design: To make learning tractable, the state of a wavelength aggregates (i) the total weighted queue backlog of its associated ONUs, (ii) the current compute quota $C_{k}\left(t\right)$, (iii) a compact histogram of slice composition, and (iv) the social sensing relationship factor $S_{rel}(t)$. Actions are restricted to migrating at most one ONU per control interval, which matches practical tuning constraints and avoids unstable oscillations.

Reward shaping: The immediate reward is defined as the incremental gain in the global objective (Equation (8)), i.e., the increase in total processing pool utility after the action, which directly reflects improved support for collaborative intelligent sensing tasks in Social IoT. This reward is augmented by $\beta \cdot S_{rel}(t)$ (where β is a tunable weight) to incentivize collaborative sensing in Social IoT. This cooperative reward encourages wavelengths to coordinate rather than compete, promoting social relationship-based collaboration. To prevent excessive re-tuning, which may disrupt real-time sensing coordination, a small switching cost is subtracted from the reward whenever an ONU migrates between wavelengths, accounting for transceiver tuning latency and control-plane overhead.

Multi-agent coordination: Each wavelength maintains its own Q-table, but all agents share the same global reward signal to encourage cooperative behavior among distributed sensors in Social IoT. The centralized controller enforces the feasibility constraints demonstrated in Equations (9)–(14) after each joint action, ensuring priority-consistent support for collaborative sensing tasks. This centralized training with decentralized execution pattern is well suited to controller-based deployments in Social IoT networks: the controller computes rewards and enforces constraints, while per-wavelength policies remain lightweight and interpretable for real-time autonomous decision-making.

In Social IoT environments, social sensing relationships are established among distributed AI-powered sensors based on relations such as ownership, co-location, co-work, and social object relations. These relationships facilitate collaborative intelligent sensing. We define the social sensing relationship factor $S_{rel}(t)\in [0,1]$ as the normalized average collaboration intensity under the wavelength at time t, computed as the mean number of active social ties per sensor divided by the maximum possible number of ties. This factor is incorporated into the state representation and reward shaping to prioritize wavelengths supporting strong collaborative sensing tasks.

According to the Bellman optimality criterion, when Equation (17) is satisfied, $V^{*}(s,a)$ is the optimal policy for the ONU [20].

(17) $$V^{*}(s,a)=\underset{a\in A}{\max}\left(V(s,a)+\gamma \sum_{s^{\prime }\in S}P_{s,a}(s^{\prime })V(s^{\prime })\right),$$

where $P_{s,a}(s^{\prime })$ is the state transition probability of the ONU from state s to s’. The Q-function is iteratively updated through interaction with the environment to maximize computational resource utility. The update equation for the Q-function is as follows:

(18) $$Q(s,a)=V(s,a)+\gamma \sum_{s^{\prime }\in S,a^{\prime }\in A}\max V^{*}(s^{\prime },a^{\prime }).$$

By interacting with the environment and iteratively updating the Q-function, the utility of computational resources is maximized. The update equation for the Q-function is as follows:

(19) $$Q(s,a)=Q(s,a)+\delta \left(V(s,a)+\gamma \sum_{s^{\prime }\in S,a^{\prime }\in A}\max Q(s^{\prime },a^{\prime })-Q(s,a)\right).$$

The Q-learning algorithm converges when updating $Q_{i}(s,a_{i})$, where δ represents the learning rate for updating Q(s,a). A higher δ leads to faster convergence, but a higher learning rate may lead to local optima rather than global optima [36]. During the learning process, balancing the relationship between exploration and exploitation is an important issue. Exploration refers to the agent choosing other unknown actions outside the known (state, action) pair distribution. Exploitation refers to the agent choosing the optimal action based on the principle of maximizing action value among all known (state, action) pair distributions. To balance exploration and exploitation, the Boltzmann distribution is typically used, with the Q-value grading function as the action probability [37].

(20) $$\phi_{i}(s,a_{i})=\frac{e^{Q(s,a_{i})/\tau }}{\sum_{a_{k}\in A}e^{Q(s,a_{k})/\tau }}.$$

If the average reward differences between actions are small, the differences in their selection probabilities are also small. If the average reward of certain actions is significantly higher than others, the probability of these actions being selected will also be higher. That is, the action with the highest reward has the highest probability of being selected, while other actions are ranked and weighted according to their Q-values. Here, *τ* is a positive parameter. The smaller τ is, the higher the probability of selecting actions with higher average rewards. The larger τ is, the more equal the selection probabilities of all actions tend to be. When *τ* trends toward 0, the algorithm tends to exploit; otherwise, it tends to explore.

Algorithm 1 presents the pseudo-code for the service priority-aware resource allocation (SPRA) algorithm based on Q-learning, where t is the maximum number of steps per episode.
**Algorithm 1:** Service priority-aware resource allocation algorithm.1: Initialize episode, wavelength assignments (pre-guided by social sensing relationship factor $S_{rel}(t)$ to promote sensor collaboration), ONU carrying states, Q-values Q(s,a) and $\phi_{i}(s,a)$ 2: for each step of an episode to t steps, do the following:  3:      Each wavelength selects action a in state s using Equation (20) with $S_{rel}(t)$
 4:      Calculate allocatable resources for wavelengths using Equation (3) 5:      Calculate current reward V(s,a) based on Equation (13), augmented by $\beta \cdot S_{rel}(t)$
 6:      The wavelength acquisition state $s^{\prime }$, $s^{\prime }$ will be set to the current wavelength state *s*, including updated $S_{rel}(t+1)$
 7:      Update Q(s,a) via Equation (19) 8:      Update $\phi_{i}(s,a)$ via Equation (20) 9: end

At the beginning of each episode, the network state is initialized. Then, according to Equation (20), action a is selected for each wavelength and executed. The reward after executing the current action is calculated according to Equation (3), and the state $s^{\prime }$ is updated to the current state s. The sum is updated to $Q_{i}(s,a)$. $\phi_{i}(s,a)$ When the number of iterations reaches t, the next episode begins.

Computational complexity: Per step, SPRA performs action selection and Q-value updates for each wavelength. In a naive tabular implementation, this is $O\left(K\cdot |A|\right)$. With the restricted action design (single-ONU migration) and compact state features, the per-step overhead is linear in the number of candidate migrations. Memory complexity is $O\left(K\cdot |A|\right)$, which can be reduced through state aggregation or function approximation for large O and L.

## 4. Simulation Analysis

To improve reproducibility, Table 2 summarizes key simulation parameters, including the number of ONUs, number of active wavelengths, processing pool capacity, service mixes, and learning hyperparameters. The parameters are selected according to typical NG-PON/CIS-RAN resource allocation scenarios and related RL studies. The mixed Poisson traffic model is adopted to emulate heterogeneous sensing arrivals from different service slices. EF traffic represents latency-critical perception tasks with relatively stringent delay requirements, AF traffic represents throughput-assured cooperative inference tasks, and BE traffic represents elastic monitoring tasks. Specifically, the slice weights EF = 3, AF = 2, and BE = 1 reflect the relative priority difference among latency-critical, throughput-assured, and elastic services. The Q-learning parameters δ = 0.1, γ = 0.9, and τ = 0.6 are commonly adopted in RL-based resource allocation studies to balance convergence stability and exploration. Unless otherwise stated, arrivals follow a mixed Poisson process per slice, and per-packet compute demands are drawn from slice-specific distributions to emulate heterogeneous processing complexity typical of collaborative intelligent sensing tasks in Social IoT environments. To reduce the influence of stochastic traffic generation and initial wavelength assignment, each simulation setting is independently repeated over 30 random seeds. The reported curves and tables present the mean values over these independent runs. During the repeated simulations, the standard deviations of the main metrics, including total utility, wavelength satisfaction, and resource utilization, are also checked to evaluate result stability. The observed trends remain consistent across different random seeds. The simulations are implemented in Python 3.6 using PyCharm 2019. All experiments are executed on a personal computer with an Intel Core i7 CPU and 16 GB RAM.

### 4.1. Performance Indicators

In collaborative sensing systems, computational resource satisfaction directly reflects inference timeliness and QoE preservation. The purpose of resource management is to improve resource utilization while ensuring QoS. Therefore, in addition to using the effectiveness of wavelength and BBU pool as evaluation indicators, in conjunction with reference [9], resource utilization and wavelength satisfaction are defined as indicators to measure system performance, as shown in Equations (21) and (22).

(21) $$u(t)=\frac{\sum_{k\in L}\min\left\{c_{l}^{k}(t),r_{l}^{k}(t)\right\}}{c_{total}},$$

where u(t) represents the proportion of computing resources currently used by the processing pool relative to the total resources, and $r_{l}^{k}(t)$ represents the computing resource requirement wavelength *k* at time *t.* $f_{l}^{k}(t)$ represents the level of fulfillment of the wavelength computing resource requirement.

(22) $$f_{l}^{k}(t)=\frac{\min\left\{c_{l}^{k}(t),r_{l}^{k}(t)\right\}-c_{k}^{\min}(t)}{r_{l}^{k}(t)-c_{k}^{\min}(t)}.$$

The adopted utility and satisfaction metrics are used as system-level proxies for sensing QoE. These metrics do not directly measure sensing accuracy, inference correctness, sensing fidelity, or application-level task success. Therefore, the reported performance gains should be interpreted as improved resource-level support for sensing QoE rather than direct evidence of improved sensing-level performance. Higher utility indicates better priority-consistent resource support, while higher wavelength satisfaction implies that latency-sensitive sensing tasks are more likely to obtain sufficient wavelength and computing resources, thereby reducing queueing delay and inference waiting time.

Furthermore, to evaluate the performance of the proposed resource allocation mechanism, comparisons are made with resource allocation schemes from [38,39,40]. Weight Ratio Resource Allocation Scheme (WRRA): First, the weight ratio of each wavelength is calculated based on the QoS of the services in each wavelength. Then, wavelengths are sorted from smallest to largest according to their weight ratio, and computational resources are allocated to wavelengths based on demand. Demand-proportional resource allocation scheme (DPRA): Resources are allocated based on the proportion of a user’s demanded resources relative to the total current network demand. For WRRA and DPRA, we implement the same minimum-compute feasibility checks as in Equations (8) and (9) to avoid infeasible allocations. WRRA allocates compute in descending order of wavelength weights until the pool budget is exhausted, while DPRA allocates proportionally to requested compute. In addition, two state-of-the-art baselines are introduced: Deep Q-Network Resource Allocation (DQN-RA) [41], which uses a three-layer fully connected neural network (ReLU activation) with experience replay and target network updates to approximate the Q-function; and Lyapunov-Based Online Optimization (Lyapunov-OPT) [42], a model-based approach using Lyapunov drift-plus-penalty to make per-slot allocation decisions with provable queue stability guarantees. In all schemes, ONU wavelength tuning is performed at the same control interval, ensuring a fair comparison in terms of reconfiguration opportunities.

### 4.2. Data Analysis

The computing resources in the processing pool are abstracted as RBs. The network has 50 ONUs, with four active wavelengths. Data packets are randomly assigned to each ONU based on service type and requirements. The current service type proportions in the network are divided into four states shown in Table 3. The weights of EF, AF, and BE are set to 3, 2, and 1, respectively. The learning rate *δ* = 0.1, and the discount factor *γ* = 0.9. The simulated service compositions emulate typical collaborative sensing scenarios in Social IoT, including perception-dominant, balanced, and monitoring-dominant workloads.

**Figure 4:**
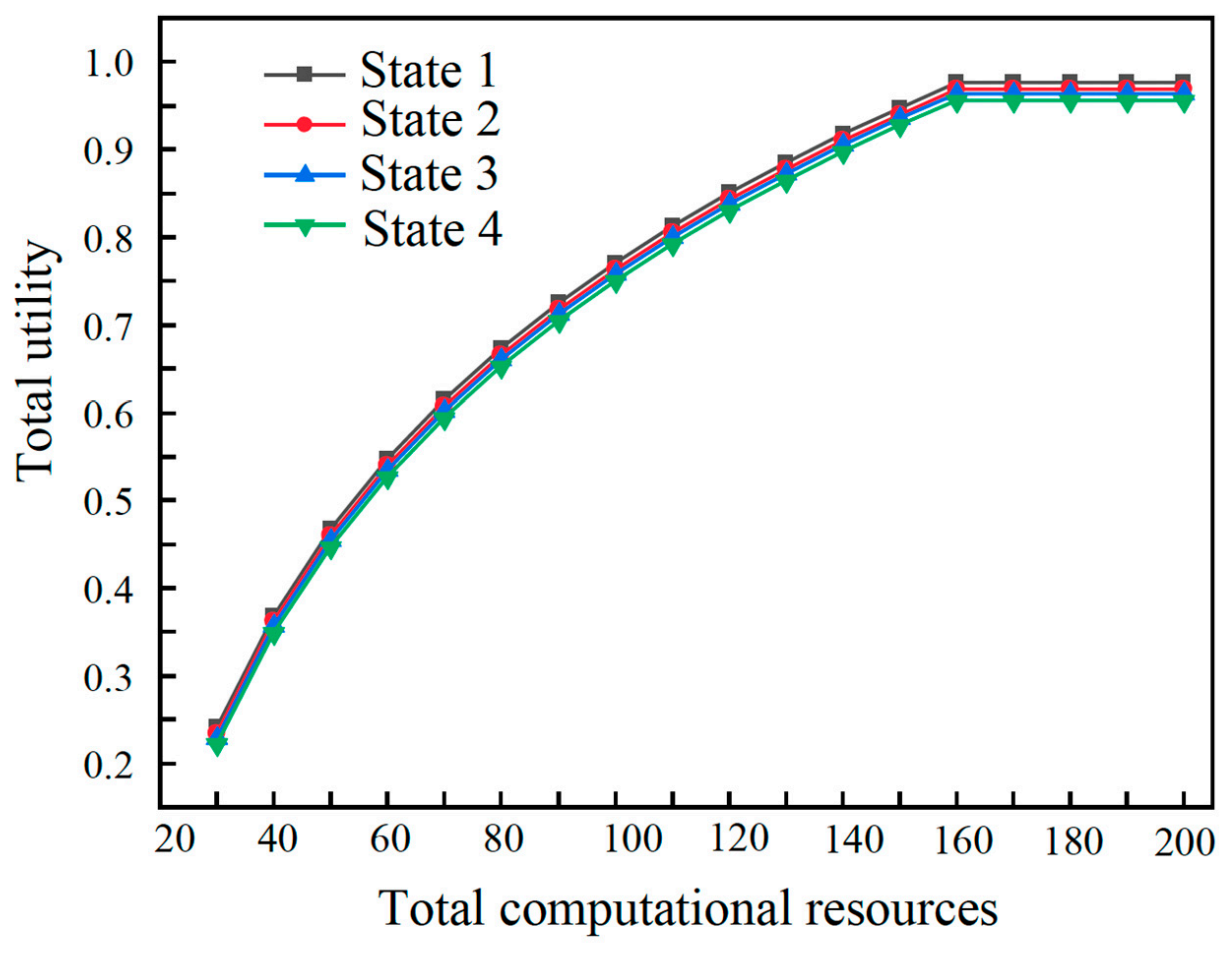
Total processing pool utility versus computational resources across four Social IoT sensing states.

Figure 4 illustrates the total processing pool utility versus total computational resources across the four network states. Total utility increases monotonically with pool capacity for all states. When resources suffice to meet all wavelength demands, allocation becomes demand-matching and utility reaches a plateau. High-weight states (e.g., State 1, EF-dominant) yield 15% higher utility than low-weight states (e.g., State 4) due to priority weighting and differentiated minimum requirements, with the gap widening by up to 30% as capacity increases.

**Figure 5:**
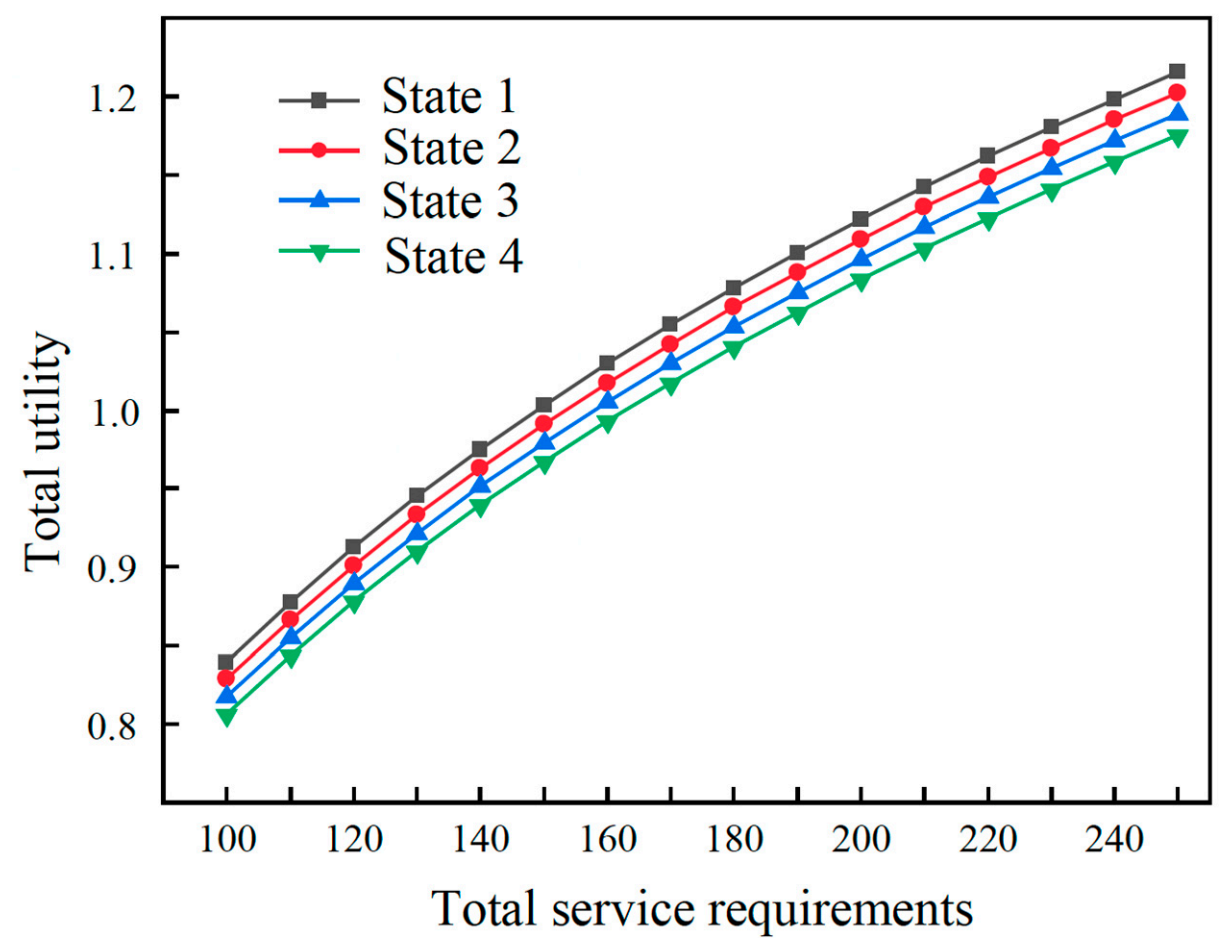
Total utility versus aggregate service demand under fixed pool capacity.

Figure 5 shows total utility versus aggregate service demand under fixed pool capacity. Utility increases nonlinearly with demand and gradually saturates, reflecting diminishing marginal returns in supporting collaborative intelligent sensing tasks in Social IoT.

**Figure 6:**
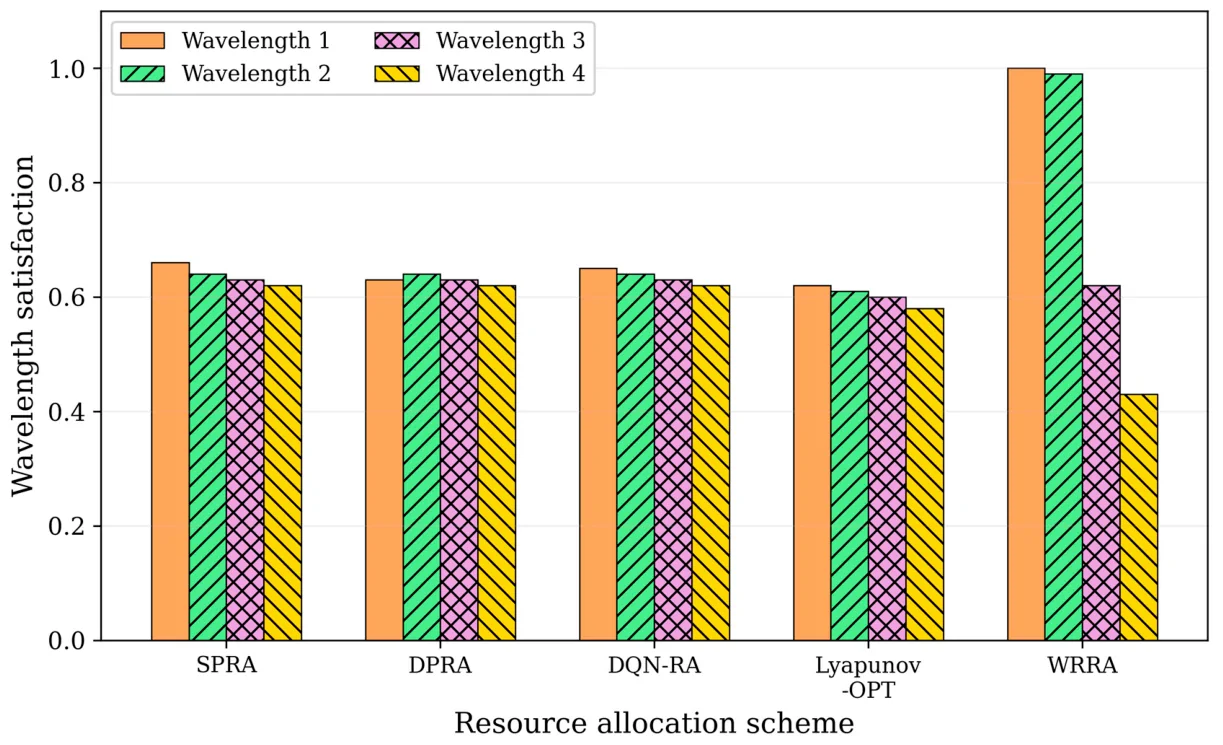
Wavelength satisfaction of SPRA, WRRA, DPRA, DQN-RA, and Lyapunov-OPT.

Figure 6 compares the wavelength satisfaction $f_{l}^{k}(t)$ of SPRA, WRRA, DPRA, DQN-RA, and Lyapunov-OPT under State 1 (EF-dominant, high-priority collaborative sensing traffic) when computational resources are insufficient. Wavelength indices are sorted in descending order of weight for SPRA and WRRA. Satisfaction for SPRA and WRRA increases with weight, with high-priority wavelengths achieving >95% satisfaction. WRRA strictly prioritizes in weight order, leaving low-weight wavelengths at <50% satisfaction under scarcity. DPRA provides uniform satisfaction but weaker high-priority protection. SPRA achieves higher average satisfaction for EF/AF slices than DPRA, demonstrating better priority-aware resource support for real-time multimodal perception and edge AI-driven inference in Social IoT. DQN-RA achieves satisfaction patterns similar to SPRA, confirming that the proposed lightweight tabular Q-learning approach attains competitive performance in the tested scenarios comparable to deep RL methods. Lyapunov-OPT provides moderate prioritization but yields lower satisfaction for high-priority wavelengths than SPRA, due to its reliance on stationary traffic assumptions that limit adaptability in dynamic Social IoT environments.

**Figure 7:**
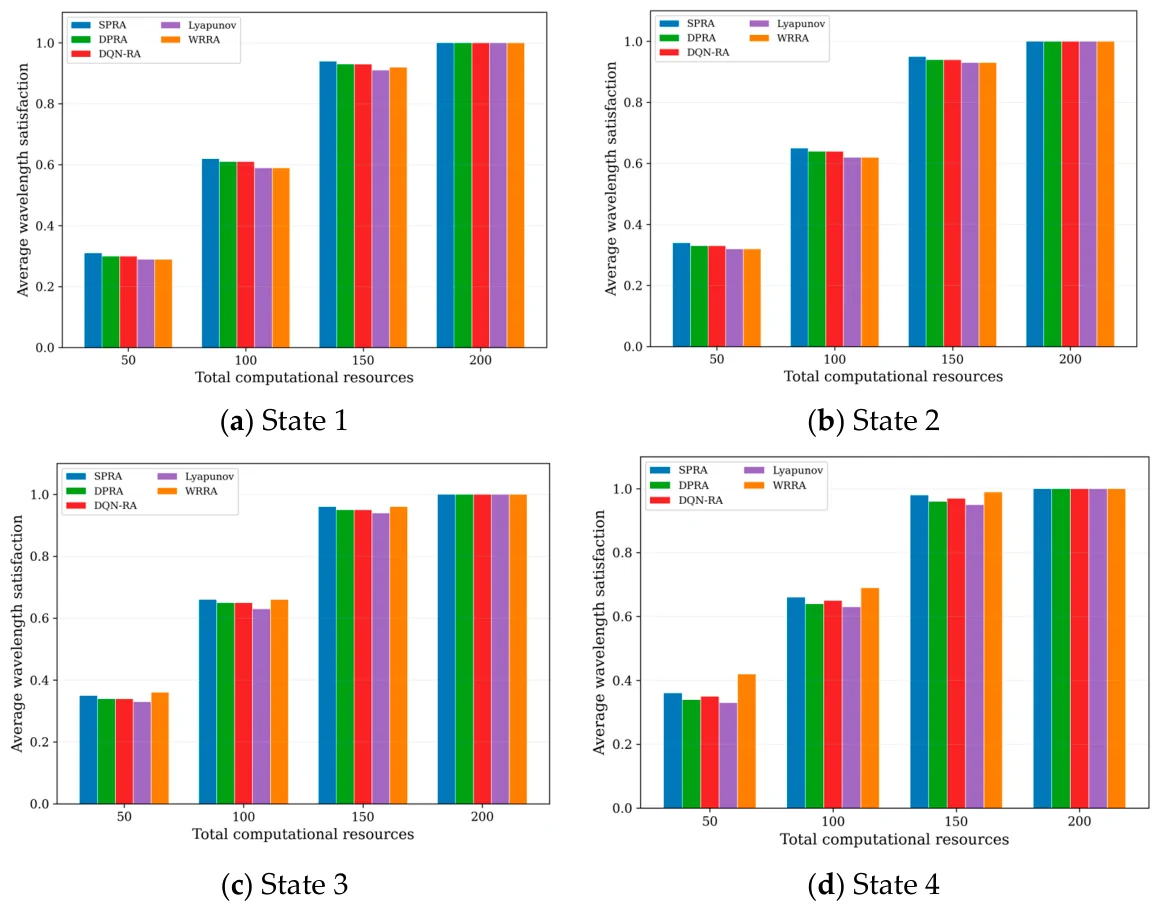
Average wavelength satisfaction of SPRA, WRRA, DPRA, DQN-RA, and Lyapunov-OPT across network states and pool capacities in Social IoT.

Figure 7 presents the average wavelength satisfaction for SPRA, WRRA, DPRA, DQN-RA, and Lyapunov-OPT across the four network states under varying total computational resources, with service demand held constant. Average satisfaction increases with pool capacity for all schemes as more resources become available to meet demands. In resource-constrained regimes, average satisfaction for SPRA and WRRA rises as the proportion of BE-type traffic increases, since elastic BE services can be accommodated more flexibly with additional capacity; WRRA exhibits greater sensitivity to traffic composition than SPRA due to its strict weight-based ordering. DPRA’s average satisfaction remains relatively stable across states, reflecting its demand-proportional allocation that distributes resources more uniformly regardless of priority mix. Under identical network states, SPRA and WRRA achieve comparable average satisfaction overall. When BE traffic dominates (e.g., State 4), WRRA shows slightly higher average satisfaction under scarcity, as its prioritization of EF services elevates the mean by protecting high-priority allocations. Conversely, as the proportion of EF traffic increases (e.g., State 1), WRRA’s average satisfaction declines relative to SPRA, highlighting SPRA’s superior balance in maintaining QoE guarantees for latency-critical collaborative intelligent sensing tasks while preserving efficiency across heterogeneous Social IoT workloads. With the addition of DQN-RA and Lyapunov-OPT, the five-scheme comparison reveals further insights. DQN-RA shows performance close to SPRA in the tested scenarios, indicating that the lightweight tabular Q-learning design can achieve competitive performance without neural-network training overhead. Lyapunov-OPT shows slightly lower average satisfaction than SPRA in the tested resource-constrained cases, particularly under resource-constrained conditions (capacity = 50 and 100), where its model-based assumptions fail to capture rapid traffic fluctuations characteristic of dynamic Social IoT environments.

**Figure 8:**
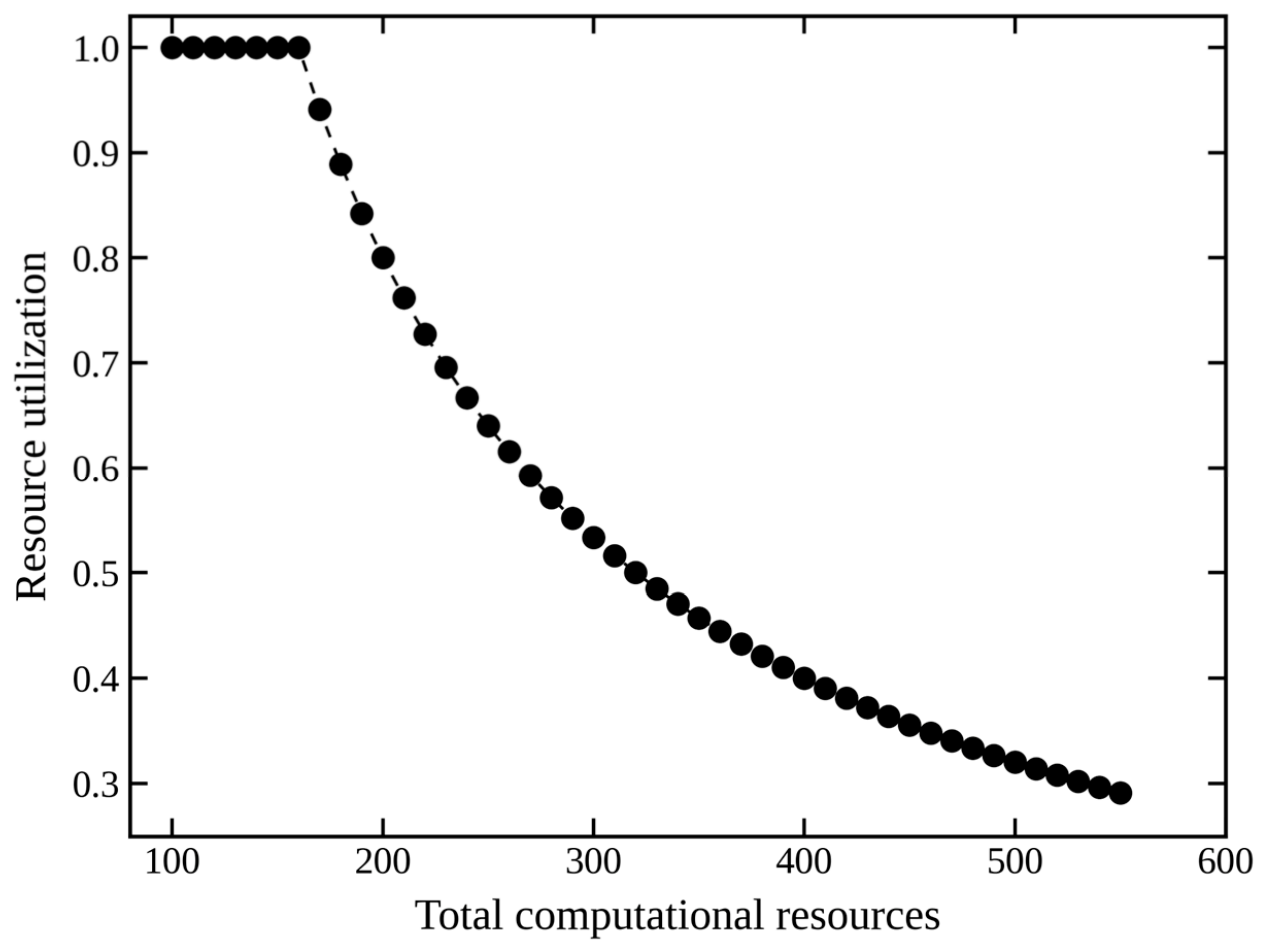
Resource utilization versus computational resources.

Figure 8 illustrates resource utilization versus computational resources under constant service demand. In severely resource-scarce conditions, utilization approaches 98–100% as all available pool resources are allocated to critical demands. Once capacity exceeds demands, utilization declines non-linearly to 30% in over-provisioned scenarios. From Figure 7, SPRA exhibits more stable and superior average wavelength satisfaction across heterogeneous traffic states compared to baselines. In balanced load conditions, SPRA achieves 10% higher resource utilization than baselines by minimizing waste through priority-aware allocation, thereby ensuring robust QoE support for high-priority collaborative intelligent sensing tasks in resource-constrained Social IoT environments.

To verify the contribution of each innovation, three ablation variants are designed: SPRA-1: Removes dynamic weight model (uses static weights). SPRA-2: Removes GNBS cross-domain formulation (single-domain optimization). SPRA-3: Replaces Boltzmann exploration with ε-greedy (ε = 0.1). Table 4 presents the performance comparison of the ablation variants. Each component contributes significantly to the overall performance in supporting collaborative intelligent sensing in Social IoT: the dynamic weight model improves total processing pool utility by 11.2% and reduces average latency by 15.3%, enhancing QoE protection for high-priority multimodal perception and edge AI-driven inference tasks; the GNBS cross-domain formulation boosts resource utilization by 9.8%, enabling more efficient coupling between wavelength tuning and compute allocation; and Boltzmann exploration accelerates convergence by 28.1% compared to ε-greedy, ensuring robust adaptation in dynamic, heterogeneous Social IoT environments. These results further demonstrate that dynamic priority modeling and cross-domain coordination are critical for preserving sensing QoE under resource constraints.

**Figure 9:**
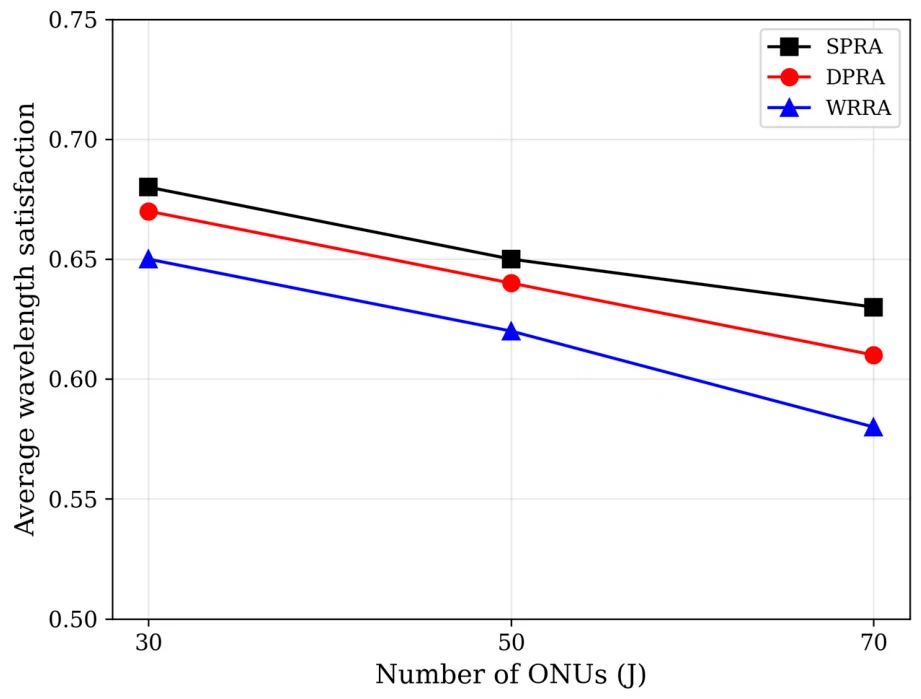
Average wavelength satisfaction versus number of ONUs.

To evaluate the scalability of the proposed framework, we conducted experiments, under varying ONU counts (J = 30, 50, 70) with pool capacity scaled proportionally, the results of which are shown in Figure 9. As J increases, average wavelength satisfaction decreases for all schemes due to intensified resource contention. However, SPRA consistently outperforms all baselines across configurations: compared to DPRA, SPRA achieves 1.5%, 1.6%, and 3.3% higher average satisfaction at J = 30, 50, and 70, respectively. The widening performance gap at a larger scale demonstrates SPRA’s superior load-balancing capability through adaptive wavelength tuning. Compared to WRRA, SPRA maintains a stable advantage of 4.6–8.6% across all tested ONU counts, further confirming the robustness of priority-aware cross-domain coordination for heterogeneous Social IoT workloads.

**Figure 10:**
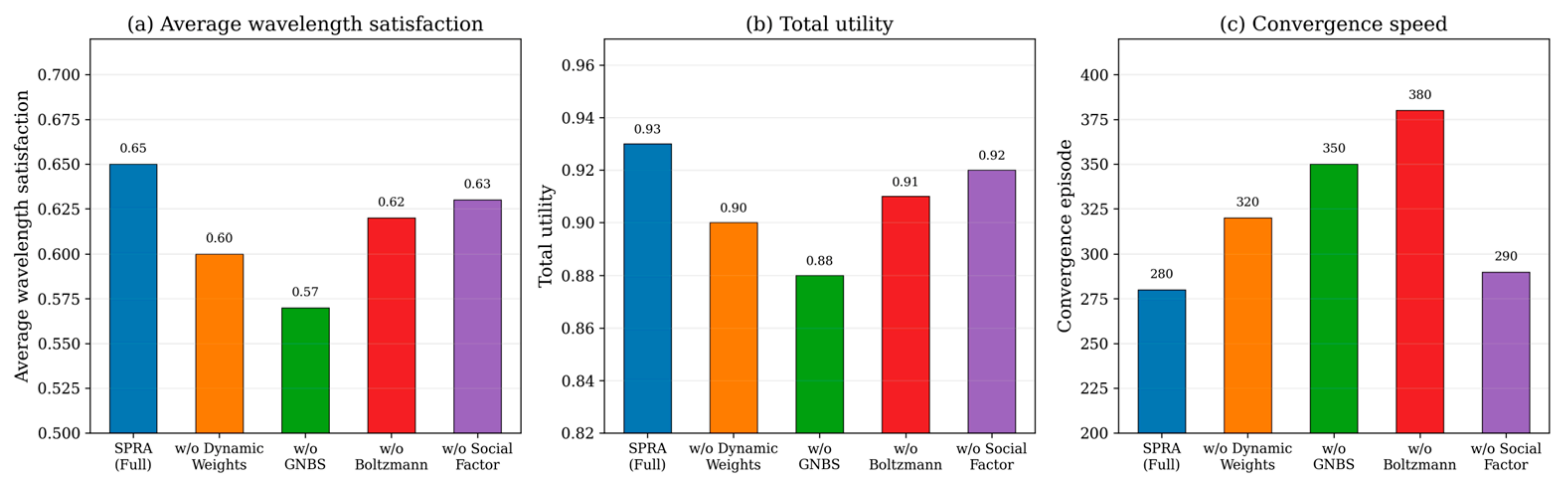
Ablation study of the proposed SPRA framework.

To further evaluate the contribution of each component, an ablation study was conducted by removing the dynamic priority weight model, GNBS-based allocation, Boltzmann exploration strategy, and social sensing relationship factor. As shown in Figure 10, the full SPRA scheme achieves the best overall performance in terms of average wavelength satisfaction and total utility, while also requiring fewer convergence episodes. Removing GNBS leads to the largest performance degradation, indicating that cooperative cross-domain allocation plays a key role in improving wavelength satisfaction and utility. Removing Boltzmann exploration mainly slows down convergence, while removing the social sensing relationship factor causes a moderate performance decrease, showing that it provides additional but relatively secondary benefits for collaborative sensing-aware resource coordination.

## 5. Conclusions

This paper proposes a priority-aware mechanism to support collaborative intelligent sensing in Social IoT via integrated NG-PON and CIS-RAN architecture. By introducing a QoE-driven dynamic weighting model, heterogeneous sensing requirements are mapped into unified priority metrics, which enables adaptive allocation of wavelength and computing resources for latency-critical multimodal perception tasks. Furthermore, a multi-agent reinforcement learning (MARL)–based strategy is employed to support cooperative and real-time resource adaptation under dynamic traffic conditions. Extensive simulation results demonstrate significant quantitative performance gains achieved by the proposed framework. Under high-priority traffic conditions, the total edge processing utility is increased by approximately 15% compared with baseline schemes. For latency-sensitive EF/AF sensing slices under resource-constrained conditions, wavelength satisfaction is improved by 8%, thereby ensuring more reliable support for critical perception services. Overall resource utilization is enhanced by approximately 10% across heterogeneous traffic scenarios. Moreover, ablation experiments indicate that the dynamic priority weighting model alone reduces the average latency by 15.3%, confirming that priority-aware modeling plays a decisive role in achieving low-latency sensing performance.

Although the proposed SPRA scheme improves system-level resource indicators in the tested scenarios, the current evaluation does not directly measure sensing-level outcomes. Future work will incorporate application-level sensing metrics and trace-driven evaluation to further verify the practical sensing benefits of the proposed framework. Future research directions include integrating edge computing by deploying edge nodes near ONUs to reduce latency for URLLC and mMTC slices, and extending the mechanism to terahertz (THz) fronthaul networks to meet higher bandwidth and lower latency demands in next-generation Social IoT applications.

## Figures and Tables

**Table 1:** Summary of key notations.

Symbol	Definition
P	Set of data packets in the buffer queue, $\left|P\right|=P$
O	Set of ONUs, $\left|O\right|=O$
L	Set of active wavelengths, $\left|L\right|=L$
$q_{i}$	Service priority weight of packet i
$w_{j}^{\mathrm{ONU}}(t)$	Weight of ONU j at time t
$w_{k}^{\mathrm{WL}}(t)$	Priority weight of wavelength k at time t
$x_{j}\left(t\right)$	ONU wavelength assignment indicator
$R_{k}\left(t\right)$	Computational resources allocated to wavelength k
$C\left(t\right)$	Total resource block (RB) budget of the processing pool
$c_{k}^{\min}\left(t\right)$	Minimum compute requirement of wavelength k
$U_{k}\left(t\right)$	Utility function of wavelength k

**Table 2:** Key simulation parameters.

Simulation Parameters	Value
Number of ONUs J	50
Number of active wavelengths K	4
Processing pool capacity	200–800 RB equivalents
Slice weights	EF = 3, AF = 2, BE = 1
Q-learning parameters	δ = 0.1, γ = 0.9, τ = 0.6
Episode length	t = 200 steps
Total episodes	1000

**Table 3:** Business share in the network.

	EF	AF	BE
State 1	60%	20%	20%
State 2	34%	33%	33%
State 3	20%	60%	20%
State 4	20%	20%	60%

**Table 4:** Ablation study results.

Variant	Total Utility	Latency (ms)	Resource Utilization (%)	Convergence Episodes
SPRA (Full)	0.92	1.23	89.5	320
SPRA-1 (Static Weights)	0.83	1.42	85.2	335
SPRA-2 (Single-Domain)	0.85	1.38	81.2	325
SPRA-3 (ε-Greedy)	0.89	1.27	88.7	445

## Data Availability

The simulation data supporting the findings of this work are available from the corresponding author upon reasonable request.
